# Estrogen receptors in pain modulation: cellular signaling

**DOI:** 10.1186/s13293-021-00364-5

**Published:** 2021-02-10

**Authors:** Qing Chen, Wenxin Zhang, Neeti Sadana, Xinzhong Chen

**Affiliations:** 1 Department of Anesthesia, Women’s Hospital, Zhejiang University School of Medicine, Hangzhou, China; 2 Department of Anesthesiology & Perioperative Medicine, Tufts Medical Center and Tufts University School of Medicine, Boston, USA

## Abstract

Sensory perception and emotional disorders are disproportionally represented in men and women and are thus thought to be modulated by different sex hormones in various conditions. Among the most important hormones perceived to affect sensory processing and transduction is estrogen. Numerous previous researchers have endeavored to demonstrate that estrogen is capable of modulating the activity of sensory neurons in peripheral and central sites in female, male, or castrated animals. However, the underlying mechanisms of its modulation of neuronal activity are somewhat unclear. In the present review, we discuss the possible cellular and molecular mechanisms involved in the modulation of nociception by estrogen.

## Introduction

Pain has been defined as “an unpleasant sensory and emotional experience associated with, or resembling that associated with, actual or potential tissue damage” by the International Association for the Study of Pain (IASP) [1]. Anatomical studies have proven that pain perception is derived from nociceptive signals produced by the activation of nociceptive neurons among the peripheral sensory nerves. The nociceptive signals are then transduced via the dorsal root ganglion (DRG) neurons, which synapse to the spinal cord dorsal horn (SDH) neurons and finally project to the thalamus and the cerebral cortex. It should be noted that estrogen receptors (ER) are widely distributed in the central nervous system, including in several nociceptive brain areas, such as the amygdala, thalamus, and anterior cingulate cortex (ACC) [2]. An fMRI study revealed that estrogen treatment could obviously change the activity of the amygdala and thalamus in rats with colorectal distension [3]. Similarly, several studies have proposed that estrogen in the ACC could significantly drive nociception-related behaviors in mice [4]. Meanwhile, pain is also modulated by a descending inhibitory pathway composed of the rostral ventromedial medulla and the 5-hydroxytryptamine (5-HT) system. Of specific interest is the role of SDH neurons in the perception and processing of sensory signals. Their neuronal interaction mechanisms have been elucidated in part by the “gate theory,” while a more comprehensive understanding is still needed [5–8]. To add to the complexity of the scenario, the endogenous opioid system is deemed the most powerful pain modulatory element and exerts its unique effects on the nociceptive system via the δ-, κ-, and μ-receptors.

Estrogen has long been found to affect the sensory and pain systems. Ovariectomized (OVX) rats or mice tend to manifest mechanical and thermal hyperalgesia [9], while supplementation with estrogen can reverse the symptoms [10]. 17-β estradiol (E2) can also alleviate neuropathic pain induced by spared nerve injury in male rats [11]. On the other hand, inhibiting estrogen receptors or knocking out ERs decreases the threshold of pain [12, 13]. These studies not only confirmed the effects of estrogen on pain modulation but also disclosed the role of ERs in mediating the function of estrogen. Estrogen can also exert its effects without the presence of ERs. The possible function of ERs in pain modulation is further supported by the discovery that ERs are ubiquitously expressed by nociceptive system neurons [2].

## Effects of ERs in different modalities of pain

ERs include two classical nuclear receptors, ERα and ERβ, that are also enriched in the cytoplasm and can be recruited into the cell membrane. The two nuclear ERs are composed of six functional domains labeled A–F, which include the N-terminal A/B domain harboring transactivation function-1 that plays a role in ligand-dependent and ligand-independent activation of the receptor, the C domain containing the DNA-binding domain, the D domain possessing recognition signals for their nuclear localization, the E domain (also known as ligand-binding domain) responsible for ligand-dependent activation of transactivation function-2 and the C-terminal F domain [14–16]. Extranuclear receptors are mainly localized in the membrane and plasma but can also be translocated into the nucleus for genetic modulation [17]. Given that the distributions of these receptors in cells vary under certain physiological or pathological conditions, their effects may differ accordingly.

Since it is ethically challenging and practically limiting to perform most pain-related experiments in humans, laboratory animal models are widely used for the study of pain. However, it may be difficult to generalize widely accepted conclusions from specific animal studies, which may produce variable results depending on the chosen experimental subject, behavioral assays, and observational measurements [18]. In this review, we focus on the most commonly used models in recent years and classify them according to the etiologies they were used to investigate, including neuropathic pain, inflammatory pain, and hyperalgesia priming [19].

### Neuropathic pain

The widely accepted definition of neuropathic pain is pain caused by a lesion or disease of the somatosensory system [8]. Most animal models are established according to this definition to mimic physiological and pathological processes in humans. Peripheral neuropathic pain is usually induced by traumatic nerve injury, such as ligation, transection, or compression of the peripheral nerves in rats or mice [20–23], whereas central neuropathic pain is mostly caused by spinal cord injury (SCI), including weight drop or contusive SCI, excitotoxic SCI, photochemical SCI, and spinal hemisection [24]. Other neuropathic pain models investigate the effects of anti-cancer drugs or chemicals used in clinic-specific diseases [19].

In the process of neuropathic pain, sexual dimorphism does exist. Vacca et al***.*** reported a sex-related difference in the development of and recovery from neuropathic pain induced by chronic constriction injury (CCI) of the sciatic nerve: male mice showed a gradual and progressive decrease in allodynic response and a complete recovery, while female mice experienced significantly longer lasting CCI-induced allodynia [25]. In addition, they further demonstrated that ERs and the agonist E2 are critical to eliminate this discrepancy between sexes [26], except for its modulatory influence depending on sex dimorphism, estrogen overall manifests a protective effect in pain modulation. As shown in the condition of central neuropathic pain, which involves both the spinal and supraspinal regions of the central nervous system, the majority of studies have demonstrated that estrogen can alleviate pain perception. For example, administration of E2 significantly increased the mechanical and thermal nociception threshold of male rats experiencing neuropathic pain induced by electrolytic spinothalamic tract lesion through suppressive effects on microglial activation or decreased glutamate levels in the thalamic ventral posterolateral (VPL) nucleus [27, 28]. In addition, estrogen signaling through ERs alleviated SCI-induced below-level neuropathic pain in part by inhibiting microglia and astrocyte activation in rats [29] and improved functional recovery from at-level neuropathic pain by inhibiting apoptosis in both males and females [30, 31]. Moreover, a recent study proved that the use of PPT, an agonist of ERα, alleviated the neuropathic pain induced by varicella zoster virus, which can cause nerve damage and inflammation mainly in the central nervous system [32].

Similar to its effect on central neuropathic pain, estrogen can alleviate nociception in most cases of peripheral neuropathic pain, partially because of the neuroprotective effects of estrogen and its receptors. However, different ER subtypes may be involved independently in mediating estrogen’s modulatory effects on neuropathic pain. One group reported that administration of a nonselective ER agonist or a selective ERα agonist had no effect in rats with spinal nerve ligation (SNL) [33] or in rats with chemotherapy-induced neuropathic pain (CINP) [34], while the use of a selective ERβ agonist effectively alleviated allodynia in both models. Additionally, contradictory results exist in a sciatic nerve CCI model of neuropathic pain in rats, and administration of E2 significantly reduced the mechanical and thermal pain threshold, which might be related to the upregulation of N-methyl-D-aspartate acid receptor 1 (NMDAR1) and its interaction with ERs in dorsal root ganglia [35]. The same pro-nociceptive effects were further confirmed by others in OVX rats using the same model [36].

### Inflammatory pain

Acute inflammation is vital for self-protection and promotion of tissue remodeling and repair, and chronic inflammation results in tissue damage and pain via pro-inflammatory mediators released at the site of inflammation [37]. The inflammatory pain model is normally induced by injecting chemicals into specific tissues or organs in rats or mice [38]. Typical chemicals used include capsaicin, mustard oil, complete Freund’s adjuvant (CFA), and formalin [39–43]. According to the chemical injection site, these inflammation models can be generally divided into three types: skin, joint, and visceral organ inflammation models [38].

In clinical cases, it has been reported that inflammatory-induced responses are more predominant in women than in men. In particular, women with endometriosis are more sensitive to pain and susceptible to chronic pelvic pain [44]. In addition, there is a consensus that fibromyalgia is more prevalent in women [45], and treatment of dysmenorrhea with hormonal contraceptives can reduce the risk of fibromyalgia [46]. These phenomena indicate that estrogen may contribute to inflammatory pain. In OVX rats with visceral pain caused by colonic inflammation, estrogen replacement was shown to facilitate nociceptive processing by increasing the activation of NMDA receptors in the spinal cord [47], which are known to contribute to hyperalgesia and neuron hyperexcitability [48]. Consistent with the above results, NMDA was shown to be coexpressed with ER in superficial dorsal horn neurons [49]. Other studies also reported that estrogen enhanced the response to noxious colorectal distention (CRD) via activation of the NMDA receptor in the spinal cord [50] or activation of the mitogen-activated protein kinase (MAPK) pathway, especially via ERα [51]. However, estrogen has also shown the opposite effect of attenuating inflammatory pain in other models. In the presence of estrogen, ERs in the DRG were reported to exert analgesic and anti-inflammatory effects in an irritable bowel syndrome (IBS) model in mice [52]. It should be noted that in addition to classical ERs, the membrane receptor GPR30 also plays a crucial role in pain modulation.

### Hyperalgesia priming

Hyperalgesia priming is a preclinical model concerning the transition from acute to chronic pain in rats, in which an increased response to pronociceptive mediators develops [53]. Activation of protein kinase Cε (PKCε) or its second messenger can induce the phenotype, which is referred to as Type I hyperalgesic priming. The phenotype can also be induced by repeated administration of an agonist at Gi-protein-coupled mu-opioid receptor or A1-adenosine receptor to the peripheral terminal of the nociceptive fibers, which is referred to as Type II hyperalgesic priming [54, 55]. It should be noted that sexual dimorphism can be observed in this model, and ERs do have a critical role in peripheral nociceptive modulation.

In Type I hyperalgesic priming, direct or receptor-mediated activation of PKCε induces priming in male rats but not in females. However, activation of the second messengers downstream of PKCε, such as the ryanodine receptor, can induce priming in both sexes [56]. Studies have shown that the difference is caused by ERα in the DRG, which prevents the induction of priming directly via PKCε but could interact with the ryanodine receptor and inositol triphosphate (IP3) receptor reciprocally to facilitate the induction of priming [57]. Similarly, sexual dimorphism has also been observed in Type II priming, especially in its maintenance process, but it is mainly dependent on GPR30 [58, 59].

Since estrogen can interact with different ERs and exert different effects, it is quite difficult to generalize its specific effects in pain modulation. However, estrogen seems to have predominantly neuroprotective effects via ERβ in neuropathic pain models and may simultaneously exert pro-inflammatory effects in inflammatory pain.

## Cellular mechanisms of ER signaling in pain modulation

Since the effects of ERs differ markedly, it is possible that the mechanisms underlying their effects also vary accordingly. The two classic nuclear ERs share a high degree of homology in their modular structure, including the DNA-binding domain and ligand-binding domain [60]. However, activation of different ERs presents different outcomes in pain modulation. For example, a study showed that ERβ agonists were effective in alleviating pain induced by chemotherapy, while the nonselective agonist 17β-estradiol and the ERα-selective agonist PPT had no effect in the same model [34]. Moreover, it has been reported that ERβ decreased the transcriptional potential of ERα, thus leading to the notion of a yin/yang balance between the two receptors in mediating estrogen function [15]. These opposing observations indicate that there may be different mechanisms of the two ERs underlying pain modulation. Consistent with this idea, the distributions of the two ERs in the nociceptive system are significantly different. In adult rat ganglia, ERα expression is restricted to small sensory neurons, while ERβ is widely expressed in different sensory neurons. In the spinal cord, whereas ERα is predominantly expressed in the superficial dorsal horn, ERβ is more highly expressed in deeper laminae [2, 61]. These results further indicate that different ER signaling pathways may be utilized in various pain modalities.

### Mechanisms of ER activation

**Fig. 1:**
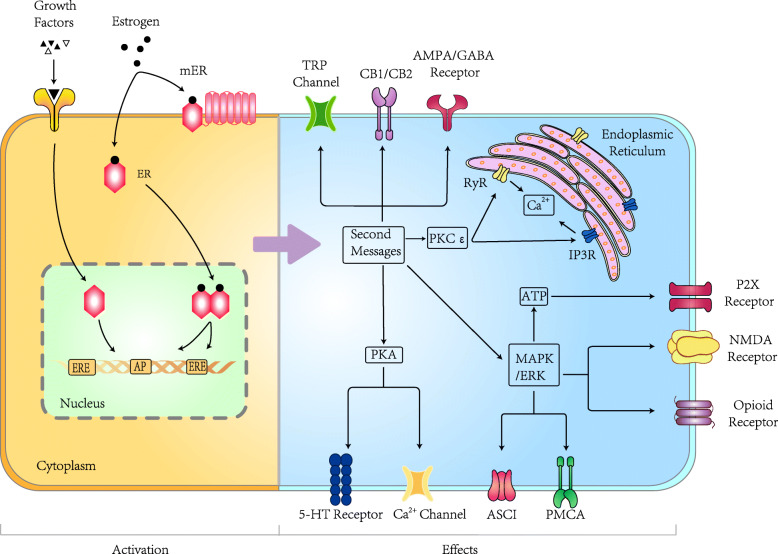
The mechanisms of ER activation and the effects of the activated ER in pain modulation

In general, there are four pathways of ER activation, as summarized in Fig. 1.

#### Classic ligand-dependent ER signaling

The two nuclear ERs are members of the nuclear receptor superfamily and mainly function via ligand-dependent mechanisms [62]. In this context, the ER is stabilized in the nucleus or cytoplasm by a complex of heat-shock proteins that mask its DNA- or ligand-binding region. When bound by the ligand in the E domain, ERs change their conformation and form homodimers that then bind to motor proteins and translocate to the nucleus, where ERs recognize with high specificity and high affinity certain DNA sequences termed estrogen response elements (EREs). The ligand-ER complex recruits transcriptional cofactors and components of the RNA polymerase II complex via AFs and subsequently regulates the transcription of target genes.

#### Membrane-initiated steroid signaling of mERs

According to the “two-step mechanism” of hormone action, it takes minutes to hours for estrogen to induce its effects. In contrast to this lengthy process, it has been reported that estrogens altered electrical activity in the uterus within several seconds [63]. During the past decades, it has become increasingly apparent that the functions of estrogen may be mediated by membrane ERs in so-called rapid/non-genomic/membrane-initiated steroid signaling [64].

#### ERE-independent signaling

Independent of EREs, ERs can interact with other transcription factors to activate transcription [65]. It has been reported that ERα activates IGF-1 and affects collagenase expression by interacting with Fos at AP-1 binding sites [66], while several genes containing GC-rich promoter sequences can be activated by an ERα-Sp1 complex [67]. It should be pointed out that E2-ERα activation of AP-1-responsive elements requires both the AF-1 and AF-2 domains of the receptor.

#### Ligand-independent signaling

It has been reported that ER function can also be modulated by extracellular signals apart from estrogens [12]. Growth factors such as epidermal growth factor (EGF) and nerve growth factor (NGF) as well as other molecules can also activate ERs [68].

Thus, a model has been proposed in which ERs travel dynamically among the membrane, cytoplasm, and nuclei to regulate both upstream non-genomic cascades and downstream genomic responses [69].

### Signaling involved in pain modulation

Nociceptors can detect numerous proinflammatory mediators, and these mediators interact with corresponding receptors on the cell surface. Subsequently, activated receptors will not only activate nociceptors to elicit nociceptive signals but also affect other receptors or ionic channels to facilitate stimulation. In the central nervous system, nociceptive information is processed and transmitted by different neurotransmitters and their respective receptors and other related molecules in pain-related signaling pathways [70]. It is likely that ERs are involved in these pathways.

#### ERα and ERβ in pain

Because they are activated by ligands in either genetic or non-genetic ways, ERα and ERβ interact with the nociceptive system to modulate pain via different cellular signaling pathways. It is commonly known that cytoplasmic calcium has a critical influence on neuronal excitability and information transmission and thus is inevitably involved in nociceptive processes [71, 72]. As described above, activation of PKCε (a subtype of PKC) promotes the release of PGE_2_ and induces sex-dimorphic hyperalgesia [56, 73]. In addition, direct activation of the ryanodine receptor (the downstream receptor of PKCε) and CaMKII α, which increases Ca^2+^ release from the endoplasmic reticulum, can also induce hyperalgesia in an ERα-dependent manner [56]. Similarly, another receptor expressed on the endoplasmic reticulum, the IP_3_ receptor, can be activated to increase Ca^2+^ release and plays a role in hyperalgesia, which involves ERα [74]. Thus, a model has been proposed in which hyperalgesia induced by the increase in Ca2^+^ released from the endoplasmic reticulum is caused by the reciprocal interaction between the ryanodine receptor and the IP_3_ receptor, which is immensely dependent on the modulation of ERα [57]. Another study discovered that the generation of IP_3_ is promoted by the interaction between ERα and group 1 metabotropic glutamate receptors (mGluR1), which activates the PKC pathway [75]. Plasma membrane calcium ATPase (PMCA) is a member of the calcium transport ATPase family and is essential for the maintenance of basal Ca^2+^ levels and the clearance of elevated intracellular Ca^2+^ [76]. As expected, knockdown of PMCA2 induced a decrease in the mechanical nociceptive threshold in rats/mice [77]. In addition, a study found that a reduction in PMCA not only abolished the analgesic effects of ERα agonists but also decreased the expression of extracellular signal-regulated kinase (ERK) in PMCA knockdown mice compared with wild-type mice, and PMCA knockdown mice presented a higher nociceptive threshold when administered an ERα agonist [78]. Thus, it may be inferred that ERα modulates pain via the MAPK/ERK pathway depending on the normal expression of PMCA2.

The endocannabinoid system, which has been proven to influence nociception, is composed of the endogenous cannabinoid ligands arachidonoylethanolamide (AEA) and 2-arachidonoylglycerol (2-AG) along with their receptors, cannabinoid receptor type 1 (CB1), which is more highly expressed in the central nervous system, and type 2 (CB2), which is more highly expressed in peripheral tissues, as well as the related enzymes involved in their synthesis and degradation [79, 80]. Cannabinoids control GABA release in the central nervous system [81], and Tabatadze et al. found that the ERα-mGluR1-IP_3_ pathway leads to AEA mobilization from postsynaptic cells to inhibit presynaptic GABA release and increase Ca^2+^ release from the endoplasmic reticulum (mentioned before) [75]. Meanwhile, it has been reported that estrogen directly modulates the expression of cannabinoid receptors; for example, the application of E2 increases the expression of CB2 in peripheral tissues [82, 83].

Purinergic receptors are widely expressed in the nervous system and have a prominent role in nociception and inflammation upon activation by adenosine triphosphate (ATP) [84, 85]. One study found that the therapeutic effect of ERβ in IBD rats was related to downregulation of the P2X3 receptor, which in turn decreased the expression of ERK and c-Fos [86]. The interaction of ERα and mGluR also inhibited the activation of the P2X3 receptor via inhibition of ATP activation [87]. Since activation of P2X3 receptors induces cation currents concomitant with the opening of voltage-gated Ca^2+^ channels, ERα has been proven to attenuate nociception by decreasing ATP-P2X3-mediated Ca^2+^ influx [88]. In addition, the expression of P2X3 receptors is apparently decreased in both ERα KO mice and ERβ KO mice [89], which confirms the correlation between the ERs and P2X3 receptors in pain modulation. TRP (transient receptor potential) ionic channels are a group of transmembrane receptors that are non-selectively permeable to cations [90]. TRP channels are widely involved in nociceptive perception and are certainly influenced by estrogen, especially in inflammatory pain conditions. The most represented subtypes of the TRP family are the capsaicin receptor TRPV1 and the noxious cold detector TRPA1 [91]. In a study investigating the expression of mRNA encoding several ion channels related to nociception, TRPV1 and TRPA1, as well as P2X3, were upregulated both in the peritoneum of women with chronic pelvic pain and in human embryonic stem cell-derived sensory neurons incubated with an ERβ agonist, which strongly indicates that ERβ is correlated with P2X3, TRPV1, and TRPA1 in nociceptive modulation [92]. Another study also showed an interaction between ERβ and TRPV1, but the activation of ERβ by E2 suppressed the function of TRPV1 in the mouse DRG [93].

Acid-sensing ion channels (ASICs) are also involved in nociceptive signaling [94]. A study found that E2 potentiated ASIC activity and that ERα acts through the MAPK/ERK and phosphatidylinositol 3-kinase/Akt (protein kinase B) pathways [95]. ERα also interacts with the NMDA receptor to mediate the effects of E2 through the MAPK/ERK pathway [51]. The NMDA receptor is a type of ionotropic glutamate receptor and is involved in the development of hyperalgesia [48, 96]. Estrogen may act on ERα to activate the phosphorylation of the NMDAR subunits NR1 and NR2 downstream of PKC and PKA in the central nervous system [97]. Given that Src can also phosphorylate NMDAR and increase its activity [98], ERα may interact with NMDAR via both Src and MAPK/ERK in the PKA or PKC pathways.

Since ERα and ERβ are nuclear receptors and can function via classic signaling mechanisms, it is highly possible that epigenetic regulation is involved in the modulation of their effects. Indeed, for example, inhibition of histone deacetylase attenuated E2-induced visceral hypersensitivity through a mechanism in which increasing binding of activated ERα and acetylated H3 to the *GRM2* promoter upregulated the expression of mGluR [99].

#### GPR30 in pain

Extranuclear ERs include membrane ER (mER)-Gαq, ERαΔ4, ER-X, and G protein-coupled ER (GPER, also known as GPR30). However, since recent studies have mainly focused on GPR30, this review also describes its effects in particular. As a member of the G protein-coupled receptor (GPCR) family, GPER30 is widely expressed in the nervous system [100]. GPR30 activates several intracellular signaling cascades via its two subunits, G_α_, which initiates ERK_1/2_ transactivation, and G_βγ_, which initiates cAMP upregulation [101], and eventually interacts with other membrane receptors or channels to exert its modulatory effects on nociception.

In several studies, GPR30 has shown a substantial influence on the opioid system, particularly via reciprocal interactions with several opioid receptors. The analgesic effects of the stimulation of the opioid receptor-like 1 (ORL1) receptor with morphine can be rapidly attenuated by administration of estrogen, which acts through membrane-initiated steroid signaling that involves GPR30 and other mERs [102]. In addition, ERK is involved in this GPR30-mediated attenuation of antinociception, since the activation of GPR30 increases the expression of phosphorylated ERK [103, 104]. Notably, G protein-gated inwardly rectifying potassium channels (GIRKs) are downstream factors of ORL1; their activation increases the response to noxious stimuli, and estrogen has been reported to exert rapid effects via GIRKs [105]. Regarding another opioid receptor, the μ-opioid receptor (MOR), Araldi et al. found via repeated administration of the GPR30 agonist DAMGO that GPR30 had regulatory effects in MOR-induced hyperalgesia [54], and GPR30 exerted these effects via Src and MAPK (mitogen-activated protein kinase)/ERK [59]. Both Src and MAPK/ERK are involved in protein kinase A (PKA) signaling and are downstream molecules of focal adhesion kinase (FAK), which is an important scaffolding protein [106]. It has been reported that kappa-opioid receptor (KOR), which shows sex dimorphic analgesic effects, can exist as a heterodimer (KOR/MOR) with MOR [107]. Not only is the expression of KOR/MOR dependent on the estrogen level [108] but also the activation of KOR/MOR by dynorphin (Dyn) via MAPK/ERK is mediated by GPR30 and other mERs [107, 109].

Estrogen also seemed to interact with the neuronal receptors that rely on cAMP generation and PKA activation in nociception. 5-HT is an important proinflammatory factor and neurotransmitter and has been reported to participate in nociceptive processes via its different receptors [110]. The activation of the 5-HT_3_ receptor has been proven to have a critical role in visceral pain, in which estrogen has significant influences [111, 112]. The rapid effects of 5-HT_3_ are definitely dependent on the activation of GPR30 and PKA [113]. The administration of sumatriptan, an agonist of 5-HT_1B/1D_, has been found not only to alleviate migraine pain [114] but also to induce hyperalgesia at the injection site [115]. Sumatriptan-induced hyperalgesia was later proven to depend on GPR30 activation as well as PKA [59]. Similar to ERα, estrogen can also rapidly modulate intracellular calcium concentration in a GPR30-dependent mechanism in which receptors on the endoplasmic reticulum and L-type calcium channels (Ca_v_1.2) on the cell membrane are cross-activated by GPR30-activated protein kinase [116, 117].

As noted above, nociceptive information is primarily processed by excitatory glutamatergic neurons and inhibitory g-aminobutyric acid-ergic (GABAergic) neurons. In addition, a-amino-3-hydroxy-5-methyl-4-isoxazole propionic acid (AMPA) receptors can be activated to facilitate excitatory transmission, while GABA_A_ receptors exert inhibitory effects in the nervous system [118]. Regarding inhibitory transmission, the vesicular GABA transporter (VGAT) is essential for the transport of GABA [32, 119, 120]. The upregulation of GPR30 can inhibit the expression of presynaptic VGAT and inhibit the function of GABA_A_ receptors in a bone cancer pain model, thus diminishing GABAergic inhibition and eventually exaggerating the perception of pain [121]. In addition, GPR30 upregulates the expression of AMPA receptors facilitating the presynaptic expression of calcium/calmodulin-dependent protein kinase II α (CaMKII α), which can facilitate excitatory transmission [121].

## Discussion

Over recent years, many studies have been dedicated to elucidating the effects of estrogen and ERs in different types of pain, and many animal models have been constructed to simulate the physiological and pathological processes of pain in humans. It is evident that estrogen has an important role in pain modulation, but its specific effects are intricate. For example, OVX rats experience hyperalgesia that can be relieved by estrogen treatment. However, estrogen also amplifies the hyperalgesia induced by activation of the ryanodine receptor in rats [56]. The contradictory results may be due to the different species, pain models, and protocols applied in different experiments. Obviously, the different types of ERs mediate different estrogenic effects. However, having been focused on the relationship between estrogen and pain over the past several years, our team highlights that the nociceptive effect of estrogen is dependent not only on its presence but also on its dynamic modulation at the system level [10], which may help explain the contradictory results regarding the mediation of nociception by estrogen.

To clarify the mechanisms of pain modulation by ERs, in this review, we summarized the different mechanisms of ER activation and several signaling pathways mediated by ERα, ERβ, and GPR30. However, it should be noted that crosstalk inevitably exists among these ERs, such as in the case of CaMKII α, which is modulated by both GPR30 and ERα [56, 121]. A physical interaction between GPR30 and ERα has also been reported [122]. In addition, in ORL1-mediated analgesia, both non-genetic (membrane ERs) and genetic (ERα and ERβ) mechanisms exist [102]. In addition to ERα-mediated Ca^2+^ release from the endoplasmic reticulum, it should be noted that GPR30 is also located in the endoplasmic reticulum and has been proven to interact with the IP_3_ receptor via PLC to upregulate the intracellular Ca^2+^ level [100, 123]. Moreover, in addition to directly engaging in the modulation of the nociceptive system, ERs can mediate various other physiological or pathological processes, thus alleviating or exacerbating pain perception in these conditions. For example, GPR30 can trigger the production of NO and the activation of eNOS, thus increasing gastrointestinal motility, which is the key factor in visceral pain induced by IBS [52]. Likewise, ERα has been found to interact with eNOS in patients with angina-induced chest pain [124].

Although many studies have proven that estrogen modulates pain via specific signaling pathways, which subtype of ER(s) is recruited under different conditions is still ambiguous. As mentioned above, growth factors induce the activation of nuclear ERs [68], and in turn, estrogen also has the ability to regulate the expression of growth factors [125]; thus, it is probable that ERs exert effects on growth factor-mediated physiological or pathological processes via the NGF/Tyrosine kinase A (TrkA, a specific receptor of NGF)-mediated pathway [126]. Indeed, a study has proven that estrogen is involved in NGF-mediated allodynia in inflamed temporomandibular joint pain [127]. Notably, since NGF is a regulator of TRPV1, estrogen also enhances allodynia induced by inflamed temporomandibular joint pain partially through TRPV1 [127]. Similarly, BDNF (brain-derived neurotrophic factor) and its specific receptor Tyrosine kinase B (TrkB) also have a role in nociception, and estrogen mediates this nociceptive process in mice [128], but the specific mechanism needs further research. Additionally, administration of E2 has been proven to upregulate the expression of anoctamin (ANO1) and TRPV1 in the trigeminal ganglia of mice with capsaicin-induced pain [129]; however, whether this E2-dependent increase in ANO1/TRPV1 is mediated by ERα or ERβ has not yet been elucidated. Given that Phosphoinositide 3-Kinase (PI3K) can be activated to associate with TRPV1 via phosphatidylinositol bisphosphate (PIP2) in NGF-mediated hyperalgesia and PI3K can be activated by E2 [130], it can be inferred that E2 may modulate NGF-dependent hyperalgesia in this pathway, but further studies are still needed to illustrate whether this pathway exists and which ER(s) are involved. Moreover, migration inhibitory factor (MIF) upregulated the expression of TRPV1 and TRPA1 at both the protein and mRNA levels, and this effect was enhanced by administration of estrogen [131], thus raising the possibility that ER also modulates pain in this MIF-mediated manner. Furthermore, TRPV1 can interact with many other receptors and systems, such as LPS and COX-2, to influence nociception [132–134], and it is unknown whether ERs participate in these processes directly or indirectly. On the other hand, NGF, TRPV1, and CB are all affected by ERs, and whether ERs mediate nociception via the effects of these pathways is also unclear. Since the nociceptive system itself is enormous, the understanding of the interaction between ERs and the nociceptive system has remained limited.

## Conclusion

As shown in the present review, there is considerable evidence that estrogens and ERs modulate pain, at least in several commonly used animal models, including peripheral and central neuropathic pain models, inflammatory pain models, and hyperalgesia priming models. Distinct signaling pathways of pain modulation involving ERs were described. However, the mechanisms by which ERs ameliorate or facilitate pain are still largely unknown. The animal models used in previous studies cannot model all types of pain or all of the effects of ERs on pain modulation. Pain perception depends on many factors, such as pain type, location of pain, level of estrogens and sex, which makes it difficult to comprehensively describe the mechanisms underlying nociception in these conditions. Further studies are warranted to investigate the function and signaling mechanisms of specific subtypes of ERs in pain modulation.

## Data Availability

Not applicable.

## Abbreviations

2-AG: 2-arachidonoylglycerol
5-HT: 5-hydroxytryptamine
AEA: Arachidonoylethanolamide
Akt: Protein kinase B
AMPA: A-amino-3-hydroxy-5-methyl-4-isoxazole propionic acid
ANO1: Anoctamin
ASICs: Transient Receptor Potential channels
BDNF: Brain-derived neurotrophic factor
CaMKII α: Calcium/calmodulin-dependent protein kinase II α
Ca_v_1.2: L-type calcium channels
CB1/2: Cannabinoid receptor type 1/2
CCI: Chronic constriction injury
CINP: Chemotherapy-induced neuropathic pain
CRD: Colorectal distention
DRG: Dorsal root ganglion
Dyn: Dynorphin
E2: 17β-estradiol
EGF: Epidermal growth factor
EREs: Estrogen response elements
ERK: Extracellular signal-regulated kinase
ERs: Estrogen receptors
FAK: Focal adhesion kinase
GABAergic: G-aminobutyric acid-ergic
GIRK: G protein-gated inwardly rectifying potassium channels
GPCR: G protein-coupled receptors
GPR30: G protein-coupled estrogen receptor
IASP: International Association for the Study of Pain
IBS: Irritable bowel syndrome
IP3: Inositol triphosphate
KOR: Kappa-opioid receptor
MAPK: Mitogen-activated protein kinase
mGluR1: Group 1 metabotropic glutamate receptors
MIF: Migration inhibitory factor
MOR: μ-opioid receptor
NGF: Nerve growth factor
NMDA: N-methyl-D-aspartate acid
ORL1: Opioid receptor-like 1
OVX: Ovariectomized
PI3K: Phosphoinositide 3-kinase
PIP2: Phosphatidylinositol bisphosphate
PKA: Protein kinase A
PKC: Protein kinase C
PMCA: Plasma membrane calcium ATPase
SCI: Spinal cord injury
SDH: Spinal cord dorsal horn
SNL: Spinal nerve ligation
TrkA/B: Tyrosine kinase A/B
TRP: Transient receptor potential channels
VGAT: Vesicular GABA transporter
VPL: Ventral posterolateral
